# Renal pressure‐flow relationship and renin activation in a porcine model comparing unilateral and bilateral renal artery stenosis

**DOI:** 10.14814/phy2.70082

**Published:** 2024-11-29

**Authors:** Benny Drieghe, Gunther van Loon, Sabrina Stuyvaert, Marc L. De Buyzere, Thierry Bové, Tine De Backer

**Affiliations:** ^1^ Heart Center University Hospital Ghent Ghent Belgium; ^2^ Dept of Internal Medicine, Reproduction and Population Medicine, Faculty of Veterinary Medicine Ghent University Ghent Belgium

**Keywords:** hemodynamic significance, renal artery stenosis, renin‐angiotensin‐aldosterone system, renovascular hypertension, transstenotic pressure gradient measurement

## Abstract

Because renal artery stenosis (RAS) often presents bilateral, we sought to investigate the renal pressure‐flow relationship and its relation to renin release, in the presence of a contralateral significant stenosis. A porcine model of graded unilateral RAS in the presence of a significant contralateral stenosis was created. The severity of the stenosis was expressed as the ratio between distal renal pressure (*P*_d_) and aortic pressure (*P*_a_). *P*_d_ and renal flow velocity were continuously measured using a combined pressure‐flow wire (Combowire®). Hemodynamic measurements and blood sampling for renin, angiotensin, and aldosterone were performed in baseline conditions and during progressive balloon inflation in the renal artery leading to stepwise 5% *P*_d_ decrements. Resistive index (RI) was computed as (1‐(End Diastolic V/Maximum Peak Systolic V))*100. A decrease of average peak flow velocity (APV) was observed when distal renal perfusion pressure decreased by 25% and was associated with activation of renin secretion. The RI decreased already for minimal changes in *P*_d_/*P*_a_ ratio. In an animal model of unilateral graded RAS in the presence of a significant contralateral stenosis, a 25% decrease in perfusion pressure results in a significant decrease in distal renal flow, causing a more pronounced upregulation of renin secretion when compared to a model of graded unilateral RAS without contralateral significant RAS.

**NEW & NOTEWORTHY**

Using contemporary technology allowing real‐time combined pressure and flow measurement in an animal model of unilateral graded RAS in the presence of a significant contralateral stenosis and contemporary, a 25% decrease in perfusion pressure results in a significant decrease in distal renal flow, causing a more pronounced upregulation of renin secretion when compared to a model of graded unilateral RAS without contralateral significant RAS.

## 1 INTRODUCTION

Renal artery stenosis (RAS) is encountered in 1%–5% of people with hypertension (Derkx & Schalekamp, 1994; Ram, 1997) and is more often found in patients with atherosclerosis in other vascular territories (Harding et al., 1992; Olin et al., 1990).

Percutaneous treatment of RAS seemed an attractive option to improve blood pressure (BP) control and/or to preserve renal function (White, 2006). However, angiographically successful stent implantation does not necessarily imply functional improvement (defined as improved BP control or improvement of renal function) (Bonelli et al., 1995; Giroux et al., 2000), despite a technical success rate exceeding 98% (Leertouwer et al., 2000).

Periprocedural complications including distal embolization (Hiramoto et al., 2005) and the presence of parenchymal disease (Rizzoni et al., 2003) might partially explain these disappointing data. Another possible explanation might be that patient selection was suboptimal, largely because a correct definition of hemodynamically significant stenosis was lacking as this is currently only based on angiographic (anatomical) criteria without physiological foundation.

A transstenotic pressure gradient is mandatory for a lesion to be considered hemodynamically significant. This is derived from Goldblatt's foundational canine experiments (Goldblatt et al., 1934), where clamping of both renal arteries reliably resulted in arterial BP elevation followed by rapid normalization. Experimental studies have shown that the renal artery lumen should be narrowed >75%–80% to induce hypertension (May et al., 1963).

Despite past attempts (Imanishi et al., 1992) to further define the hemodynamic relevance of RAS, a critical value for the transstenotic pressure gradient to activate the renin‐angiotensin‐aldosterone system (RAAS) has not been clearly defined.

Using contemporary technology allowing real‐time combined pressure and flow measurement in a porcine model of unilateral progressively graded RAS, we documented that a 25% decrease in perfusion pressure results in a significant decrease in distal renal flow, causing upregulation of renin secretion (Drieghe et al., 2023).

However, RAS is frequently bilateral. In a series of 1235 screening abdominal aortic angiograms, 11% of patients displayed unilateral RAS and 4% had bilateral RAS defined as ≥50% stenosis (Harding et al., 1992). In 90 high‐risk veterans referred for cardiac catheterisation, a selective renal angiography revealed unilateral RAS ≥50% in 28% of patients with 16% having a more severe unilateral stenosis of ≥70%. Bilateral RAS ≥50% was observed in 10% and 6% had bilateral RAS ≥70% (Aqel et al., 2003). Summarizing the data from 26 studies including 30,092 patients undergoing coronary angiography, Messerli and coworkers quoted that 20.3% of all patients with atherosclerotic RAS had significant bilateral RAS, defined as ≥50% luminal narrowing (Messerli et al., 2011). An autopsy study on 5194 patients concluded that RAS was present in 4.3%. In diabetic patients, this increased to 8.3%. Bilateral RAS was found in 43% of patients with diabetes and RAS, as opposed to 30% in nondiabetic patients with RAS (Sawicki et al., 1991). De Mast and Beutler, (2009) compiled data on RAS prevalence in high‐risk groups. In all groups, a RAS prevalence of 15.4% was observed; bilateral RAS was present in 4.2%.

Experimental data on this subject are scarce. Animal models since Goldblatt's foundational experiment invariably consist of clipping one renal artery while preserving (two kidney, one clip; so called “2K1C”) or removing the contralateral kidney (one kidney, one clip; so called “1K1C”). For a model to be representative for bilateral RAS, one would need a two kidney, two clip (“2K2C”) model. Yamasaki, (1987) performed neurohumoral measurements in a rabbit 2K2C model, however without hemodynamic data on pressure and flow.

Therefore, we sought to investigate which pressure gradient is required in bilateral RAS to (further) activate RAAS and promote renin secretion.

## 2 METHODS

### 2.1 Study population and description of the experiment

We performed the index study (Drieghe et al., 2023) in 16 female landrace pigs (RA‐SE Genetics, Lokeren, Belgium) of 40–50 kg. Sample size calculation was based on “differences of means with comparable standard deviation (SD)” applied to the renin levels during the experiments. For a hypothesized baseline renin level of 40 (SD25) pg.mL^−1^ and at a pre‐test hypothesized perfusion pressure decrease of 25% with renin levels hypothesized at that time of 65 (SD25) pg.mL^−1^, the calculated sample size was 16 pigs. The study complied with the standards of “the guide for the care and use of laboratory animals” published by the National Institute of Health (National Research Council (US) Institute for Laboratory Animal Research, 1996) and was approved by the institutional ethics committee for animal research (Ghent University Hospital, ECD 12/11).

After premedication with intramuscular tiletamine and zolazepam (Zoletil®, Virbac, Barneveld, The Netherlands), in a combined solution with xylazine (Xyla‐ject®, Dopharma, Raamdonksveer, The Netherlands) 2% (0.2 mL.kg^−1^), animals were anesthetized using intravenous propofol (Propolipid®, Fresenius Kabi, Schelle, Belgium) 3 mg.kg^−1^, sufentanil (Sufenta®, Piramal, Morpeth, United Kingdom) 0.005 mg.kg^−1^, and rocuronium bromide (Esmeron®, MSD, Sint‐Lambrechts‐Woluwe, Belgium) 1 mg.kg^−1^. Endotracheal intubation was performed and anesthesia was maintained with sevoflurane (Sevorane®, AbbVie, Wavre, Belgium) 2.5% using the AnaConDa system (Sedana medical AB, Sundbyberg, Sweden).

We obtained arterial access with an 8F sheath via surgical cut‐down of the right carotid artery. Next, a 6F sheath was introduced in the right and left internal jugular vein via the Seldinger technique. We positioned a 6F Amplatz left (AL1) diagnostic catheter in both renal veins for selective blood sampling. All hemodynamic measurements were performed in the right renal artery for standardization.

A model of unilateral graded RAS was created by progressively inflating a balloon 1 mm smaller than the lumen of the renal artery. A short stent was first deployed at the ostium of the right renal artery over a standard 0.014″ guidewire (Balance Middleweight Universal®, Abbott Vascular, Santa Clara, CA, USA) to enable and maintain a stable position of the balloon. Aortic pressure (*P*_a_) was measured through an 8F Judkins Right guide catheter. A combined 0.014″ pressure and flow wire (Combowire®, Volcano Corporation, Rancho Cordova, CA, USA) positioned at least 4 cm distal to the stented segment to measure distal renal pressure (*P*_d_) and Doppler flow signals. The wire was slowly rotated while monitoring the power spectrum and the auditory Doppler signal to optimize the spectral waveform.

We chose to use the ratio between *P*_d_ and *P*_a_ rather than the absolute distal pressure to minimize variations due to excessive changes in systemic arterial BP.

**FIGURE 1:**
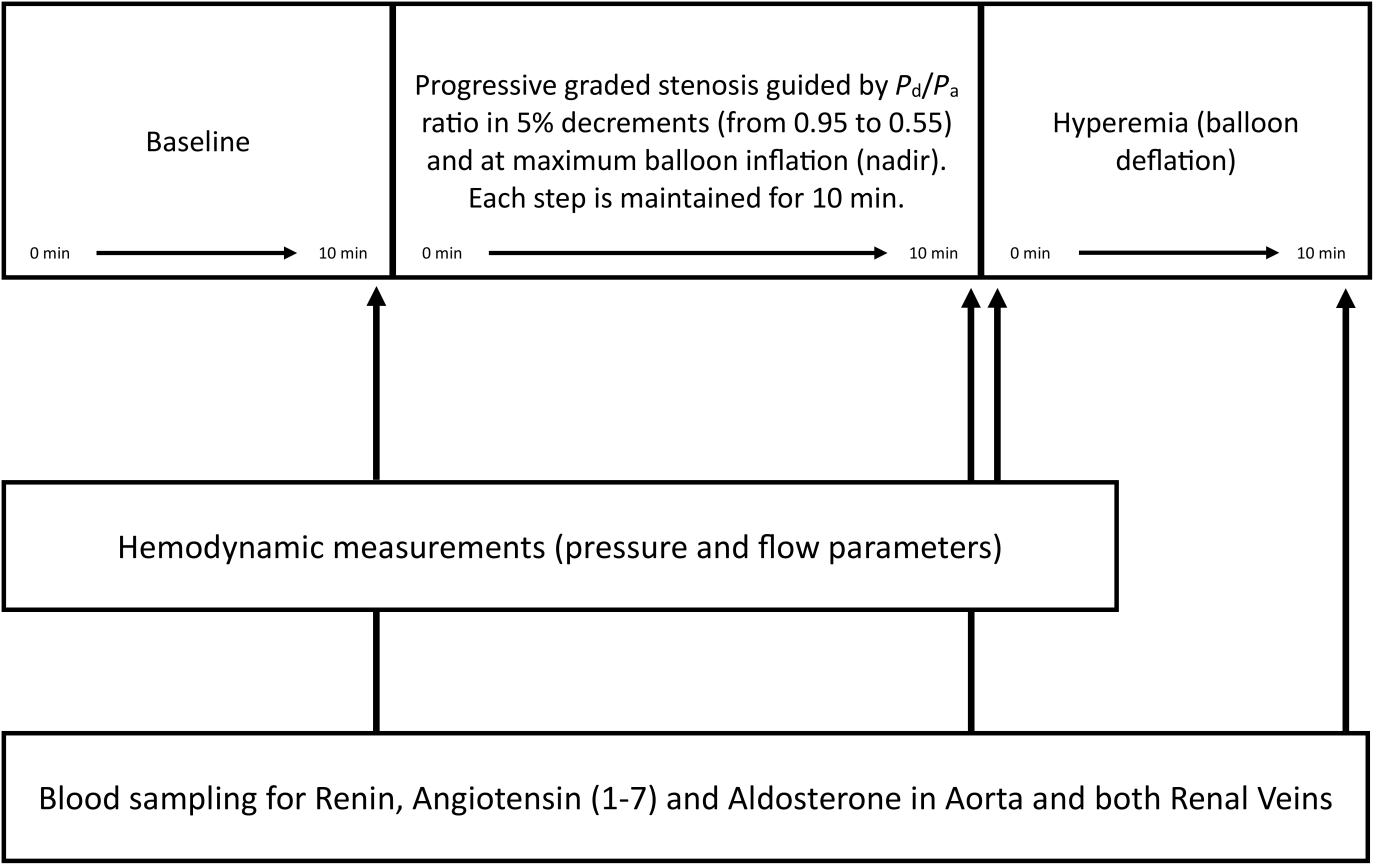
Schematic of the experiment. Top part of the figure depicts the different conditions of the experiment. Each step of progressive balloon inflation is indicated by its ratio between distal renal (*P*_d_) and proximal aortic (*P*_a_) pressure‐ each step is maintained for 10 min. Nadir indicates the lowest *P*_d_/*P*_a_ ratio reached at balloon inflation at rated burst pressure. Hyperemia is reached after balloon deflation. Hemodynamic recordings are obtained at the end of each 10 min interval at the different conditions mentioned above, except at hyperemia where data are recorded 10 s after balloon deflation. Blood sampling was performed at the end of each 10 min interval at different conditions mentioned above.

Hemodynamic measurements and blood sampling were performed at baseline. The balloon in the right renal artery was progressively inflated to obtain a distal pressure decrement of 5% per step. We maintained each step for 10 min at steady‐state conditions. At the end of each step, we obtained hemodynamic measurements and blood samples in both renal veins and aorta. As each tube contained 4 mL of blood, a total of 12 mL blood was collected at each step. A total blood volume of 144 mL was collected as the experiment consisted of 12 steps (Figure 1). This was substituted by saline solution to avoid hypovolemia. Ten mL of blood was aspirated from the catheters before collecting the actual sample, which was readministered after sampling. We aimed to reach a *P*_d_/*P*_a_ ratio of at least 0.60. The balloon was inflated up to its rated burst pressure to obtain the lowest *P*_d_/*P*_a_ ratio for 10 min (“nadir”). Finally, the balloon was deflated, and the same hemodynamic measurements were performed and blood samples were obtained during the reactive hyperemia (Figure 1).

Thereafter, a left lumbotomy was performed, exposing the left renal artery; two vascular clips were juxtaposed (to create a 2K1C model) and the wound was closed in layers as per standard procedure.

Adequate analgesia with intramuscular administration of buprenorphine 0.01–0.03 mg.kg^−1^ every 6–8 h, (Temgesic®, Indivior Europe Limited, Dublin, Ireland) and carprofen 4 mg.kg^−1^. (Rimadyl®, Zoetis, Louvain‐la‐Neuve, Belgium) was provided.

**FIGURE 2:**
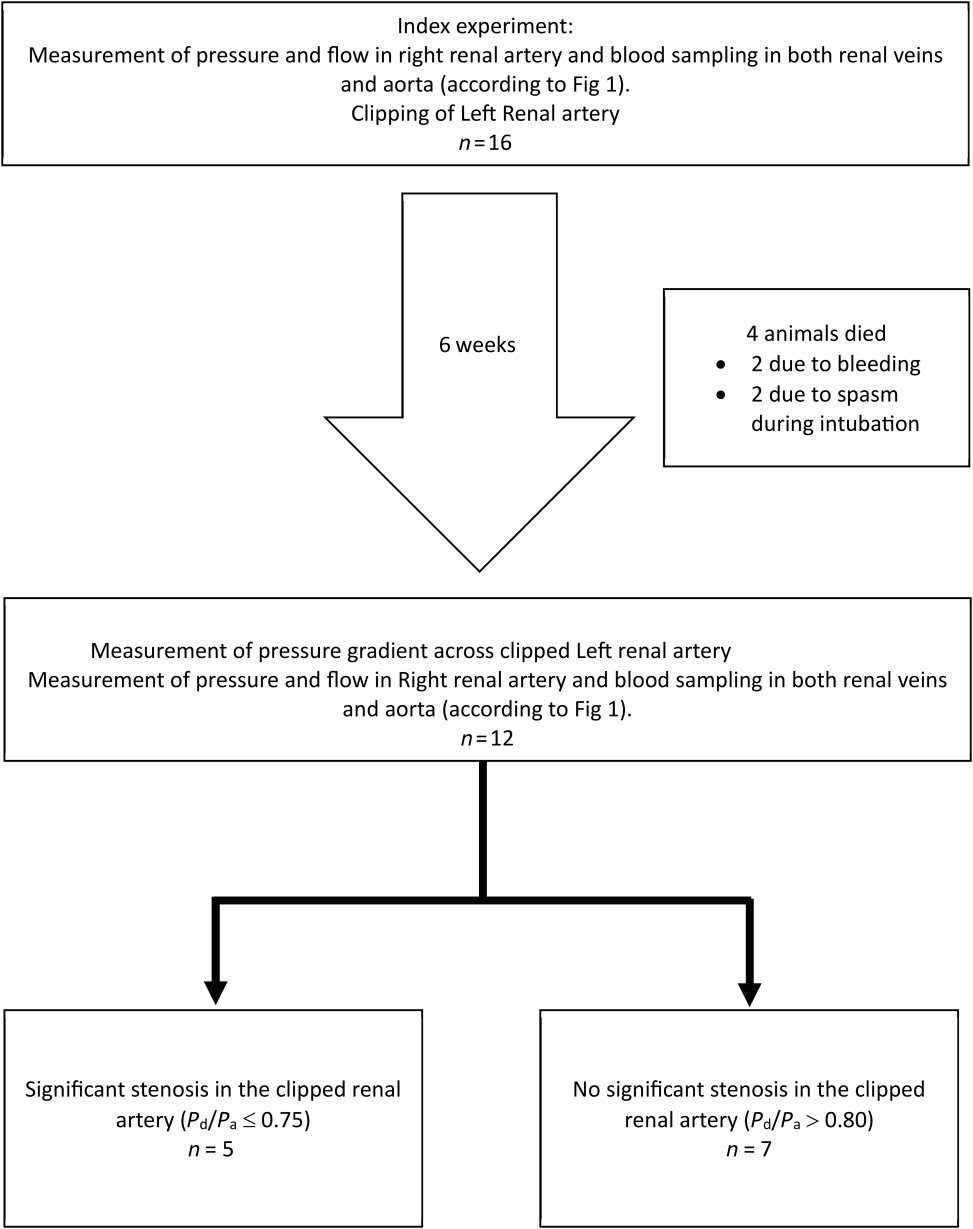
Study overview. *P*_d_/*P*_a_: Ratio between distal renal and aortic pressure. After 6 weeks, five pigs developed a significant stenosis in the clipped left renal artery, defined as a *P*_d_/*P*_a_ ratio ≤0.75, while seven animals had a *P*_d_/*P*_a_ ratio >0.80, hemodynamically not significant.

Six weeks after the index experiment, the animals were anesthetized using the earlier‐described protocol. Renal angiography was performed using a 6F Judkins Right catheter and the transstenotic pressure gradient was measured in the clipped renal artery. A significant RAS, defined as a *P*_d_/*P*_a_ ratio ≤0.75, was seen in five animals. In these animals, the above‐described protocol was repeated in the nonclipped (right) renal artery (Figure 2).

Finally, the animals were euthanised with the intravenous administration of embutramide 200 mg, mebenzoniumiodide 200 mg, tetracainehydrochloride 5 mg, and dimethylformamide 1 mg (T61) at a dose of 0.3 mL.kg^−1^.

### 2.2 Hemodynamic measurements

We measured the following renal flow parameters: average peak flow velocity (APV, cm.s^−1^), maximum peak flow velocity (MPV, cm.s^−1^) and end‐diastolic flow velocity (EDV, cm.s^−1^). Renal resistive index (RI) was computed from MPV and EDV as (1‐EDV/MPV)*100.

Measurements were obtained in baseline conditions (after successful deployment of a stent) and during each step of controlled graded RAS, quantified by *P*_d_/*P*_a_ ratio, progressively decreasing in 5% decrements. Final hemodynamic measurements were obtained 10 s after complete balloon deflation (Figure 1).

### 2.3 Neurohumoral measurements

Blood samples were immediately stored on ice. Plasma was separated during a 10 min centrifuge at 3000 g at 4°C, subsequently snap‐frozen and stored at −80°C.

We performed all analyses in triplicate. We used porcine ELISA tests for renin (catalog number E07R0022), angiotensin (catalog number E07A0206), and aldosterone (catalog number E07A0774) to assess RAAS activation (Blue‐Gene technology, Shangai, China).

### 2.4 Statistical analysis

SPSS 26 (SPSS, Chicago, Illinois) was used to perform all analyses. Data are expressed as mean values ± SD. A Wilcoxon signed rank test was used to compare baseline values and values at the prespecified levels of progressive balloon inflation. A Kruskal‐Wallis test was used to compare renin samples from baseline experiment to those 6 weeks after clipping.

A *p* value ≤0.05 was considered for statistical significance.

## 3 RESULTS

### 3.1 Physical characteristics

Weight of the pigs increased from an average of 40 kg to an average of 80 kg 6 weeks after the index experiment (due to normal growth, not due to obesity). In the five animals that developed a significant RAS, defined as a *P*_d_/*P*_a_ ratio ≤0.75, mean renal artery diameter increased from 5.8 ± 0.2 mm to 6.2 ± 0.3 mm. Mean *P*_d_/*P*_a_ ratio across the clipped artery in these pigs was 0.67 (range 0.50–0.75).

### 3.2 Comparison of baseline parameters

**FIGURE 3:**
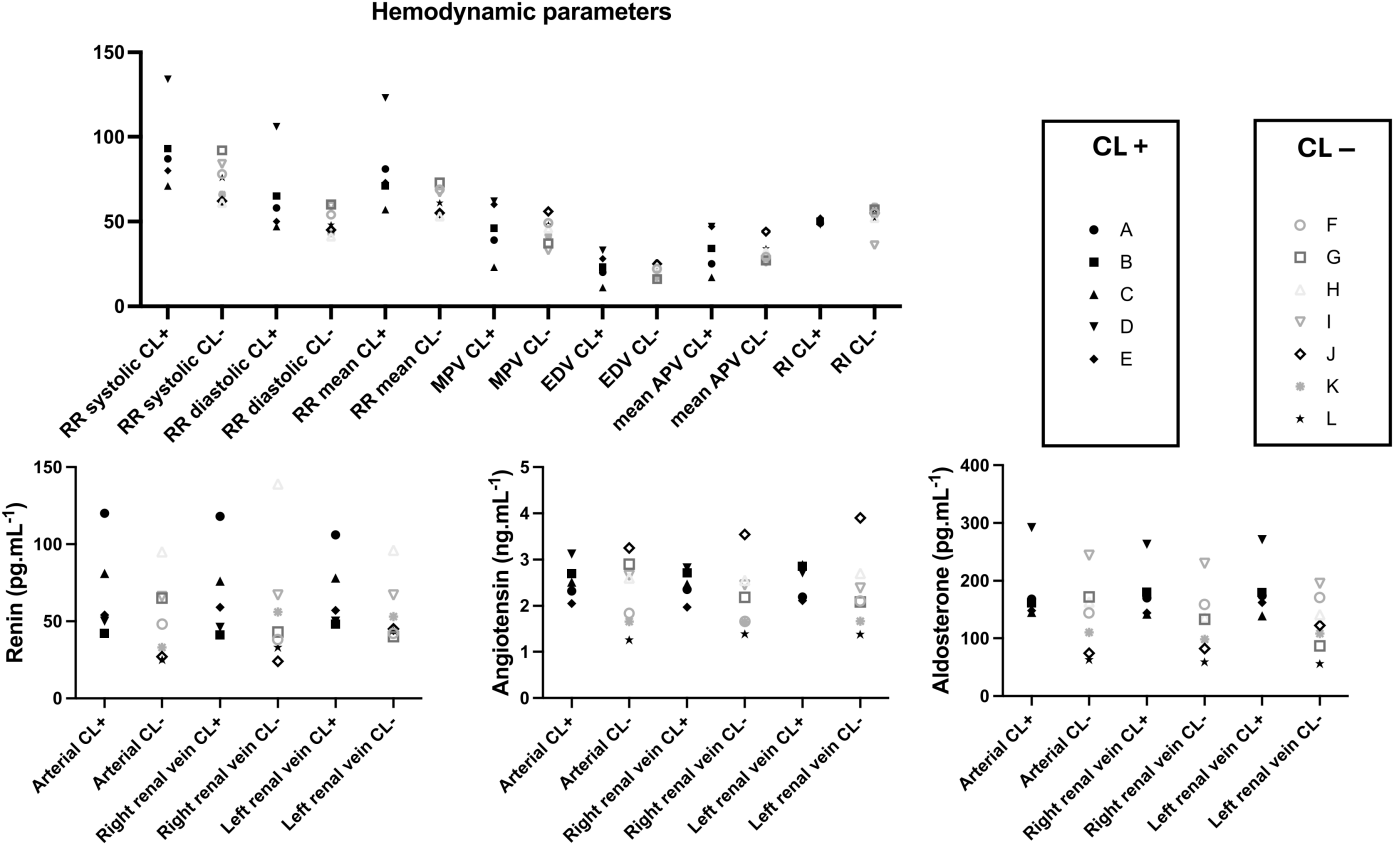
Hemodynamic and neurohumoral parameters 6 weeks after clipping of the left renal artery. The graph depicts the individual parameters from the five pigs that developed a significant stenosis after clipping (contralateral stenosis, parameter is indicated with suffix “CL +”, labeled A to E) to those from the seven pigs that did not develop a stenosis after clipping (no contralateral stenosis, parameter is indicated with suffix “CL−”, labeled F to L). Each parameter for an individual animal (A–L) is represented by its corresponding symbol. Top half depicts hemodynamic parameters on the *x* axis. For blood pressure (BP), systolic, diastolic, and mean BP are shown. Furthermore, maximum peak velocity (MPV), EDV and mean average peak velocity (APV) and resistive index (RI) are presented. Parameters are presented pairwise, first the parameter from the animals that developed a significant stenosis 6 weeks after clipping (suffix “CL+”) and next to it the same parameter from the animals that did not develop a significant stenosis after clipping (suffix “CL−”). The *y* axis should be read in mmHg for BP values, in cm.s^−1^ for MPV, EDV and mean APV and without unit for RI. Bottom half compares renin (left), angiotensin (middle), and aldosterone (right) levels from the five pigs that developed a significant stenosis after clipping (contralateral stenosis, labeled with suffix “CL+”) to the hormone concentrations from the seven pigs that did not develop a stenosis after clipping (no contralateral stenosis, labeled with suffix“CL−”). From left to right on the *x* axis, samples obtained in the aorta (“arterial”), right renal vein and left renal vein are shown. Parameters are similarly presented pairwise, first the parameter from the animals that developed a significant stenosis 6 weeks after clipping (CL+) and next to it the same parameter from the animals that did not develop a significant stenosis after clipping (CL−). Units on the *y* axis are expressed in pg.mL^−1^ (for renin and aldosterone) or in ng.mL^−1^ (for angiotensin).

In the animals who had developed a significant RAS 6 weeks after clipping, nonsignificant higher BP values and renin levels were observed compared to those that did not exhibit a hemodynamically significant RAS after clipping (Figure 3).

### 3.3 Effect of significant unilateral RAS on BP

**FIGURE 4:**
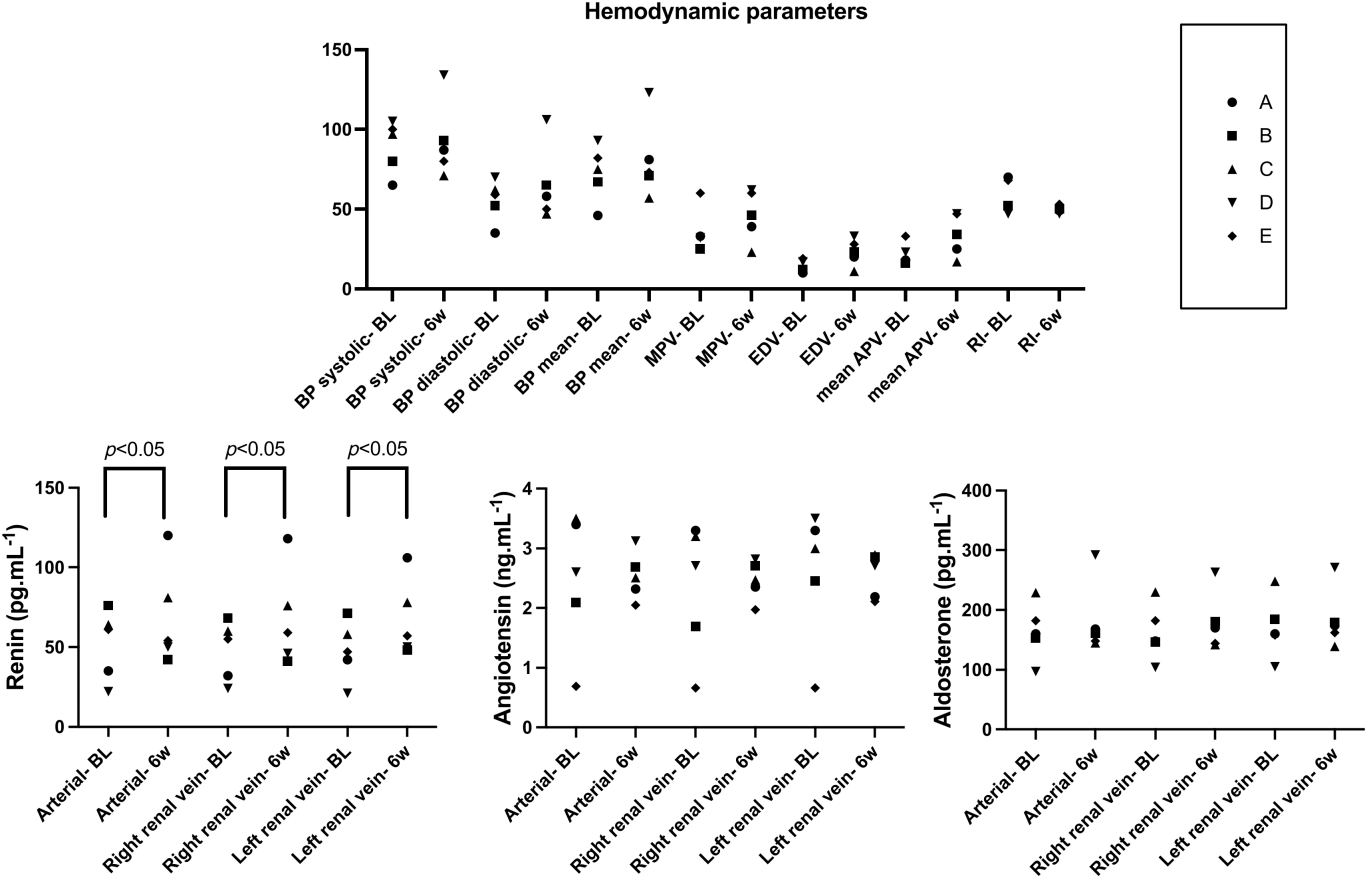
Hemodynamic and neurohumoral parameters at baseline and 6 weeks after clipping of the left renal artery in the five pigs that developed a significant renal artery stenosis (RAS) after clipping. Comparison of hemodynamic and neurohumoral parameters at baseline (labeled with suffix “BL”) and 6 weeks after clipping of the left renal artery (labeled with suffix “6w”) in the five pigs that developed a significant RAS after clipping of the left renal artery. For the definition of hemodynamic stenosis, we refer to the methods section. A–E, Each represent the parameter of an individual animal, each letter refers to the same animal in the Figure. *p* Values are mentioned when significant. Top half depicts hemodynamic parameters on the *x* axis. For blood pressure (BP), systolic, diastolic, and mean BP are shown. Furthermore, maximum peak velocity (MPV), end‐diastolic velocity (EDV) and mean average peak velocity (APV) and resistive index (RI) are depicted. The *y* axis should be read in mmHg for BP values, in cm.s^−1^ for MPV, EDV, and mean APV and without unit for RI. Parameters are presented pairwise, first the parameter at baseline (suffix “BL”) and next to it the same parameter 6 weeks after clipping (suffix “6w”). Bottom half compares renin (left), angiotensin (middle), and aldosterone (right) levels at baseline conditions (suffix “BL”) and 6 weeks after clipping of the left renal artery in the five pigs that developed a significant stenosis after clipping (suffix “6w”). From left to right on the *x* axis, samples obtained in the aorta (“arterial”), right renal vein and left renal vein are shown. Units on the *y* axis are expressed in pg.mL^−1^ (for renin and aldosterone) or in ng.mL^−1^ (for angiotensin). Parameters are presented pairwise, first the parameter at baseline (suffix “BL”) and next to it the same parameter 6 weeks after clipping (suffix “6w”).

Baseline mean arterial BP (72.6 ± 17.7 mmHg) showed a nonsignificant increase to 83.5 ± 28.2 mmHg 6 weeks after clipping of the left renal artery. A similar nonsignificant increase was observed for systolic and diastolic BP (resp from 89.4 ± 16.6 mmHg to 93.0 ± 28.1 mmHg and 55.6 ± 13.2 mmHg to 65.3 ± 27.6 mmHg) (Figure 4).

### 3.4 Effect of significant unilateral RAS on *contralateral* baseline renal flow parameters

Baseline maximum peak flow velocity (MPV) increased from a baseline value of 35.0 ± 14.5 cm.s^−1^ to 46.0 ± 18.5 cm.s^−1^ 6 weeks after clipping. Baseline end‐diastolic velocity (EDV) increased from 14.0 ± 3.8 cm.s^−1^ to 23.0 ± 9.6 cm.s^−1^.

Average peak velocity (APV) increased from 21.4 ± 7.0 cm.s^−1^ to 34.0 ± 15.4 cm.s^−1^ 6 weeks after the index experiment.

Baseline RI decreased from 57.8 ± 10.5% to 50.3 ± 3.0% 6 weeks after creating a contralateral RAS (Figure 4).

None of these changes reached statistical significance.

### 3.5 Effect of significant unilateral RAS on neurohumoral parameters

A significant rise in renin levels was observed comparing baseline values to those 6 weeks after the index experiment. Arterial, right and left venous renin levels increased from 51.6 ± 22.2, 47.9 ± 19.1 and 47.9 ± 18.8 pg.mL^−1^ to 69.5 ± 31.7, 68.0 ± 30.9 and 67.7 ± 24.7 pg.mL^−1^ respectively; *p* < 0.05.

Baseline Angiotensin and Aldosterone levels remained unchanged 6 weeks after induction of a significant RAS (Figure 4).

### 3.6 Effect of graded RAS on renal flow parameters in the presence of *contralateral* significant RAS

Maximum peak flow velocity decreased markedly (from 46.0 ± 18.5 to 30.0 ± 7.4 cm.s^−1^, *p* = 0.05) at a *P*_d_/*P*_a_ ratio of 0.80. End‐diastolic velocity (EDV) remained relatively constant up to a 20% decrease in perfusion pressure. A significant decrease (from 23.0 ± 9.6 to 3.0 ± 0.8 cm.s^−1^, *p* < 0.05) in EDV was observed at maximum balloon inflation.

**FIGURE 5:**
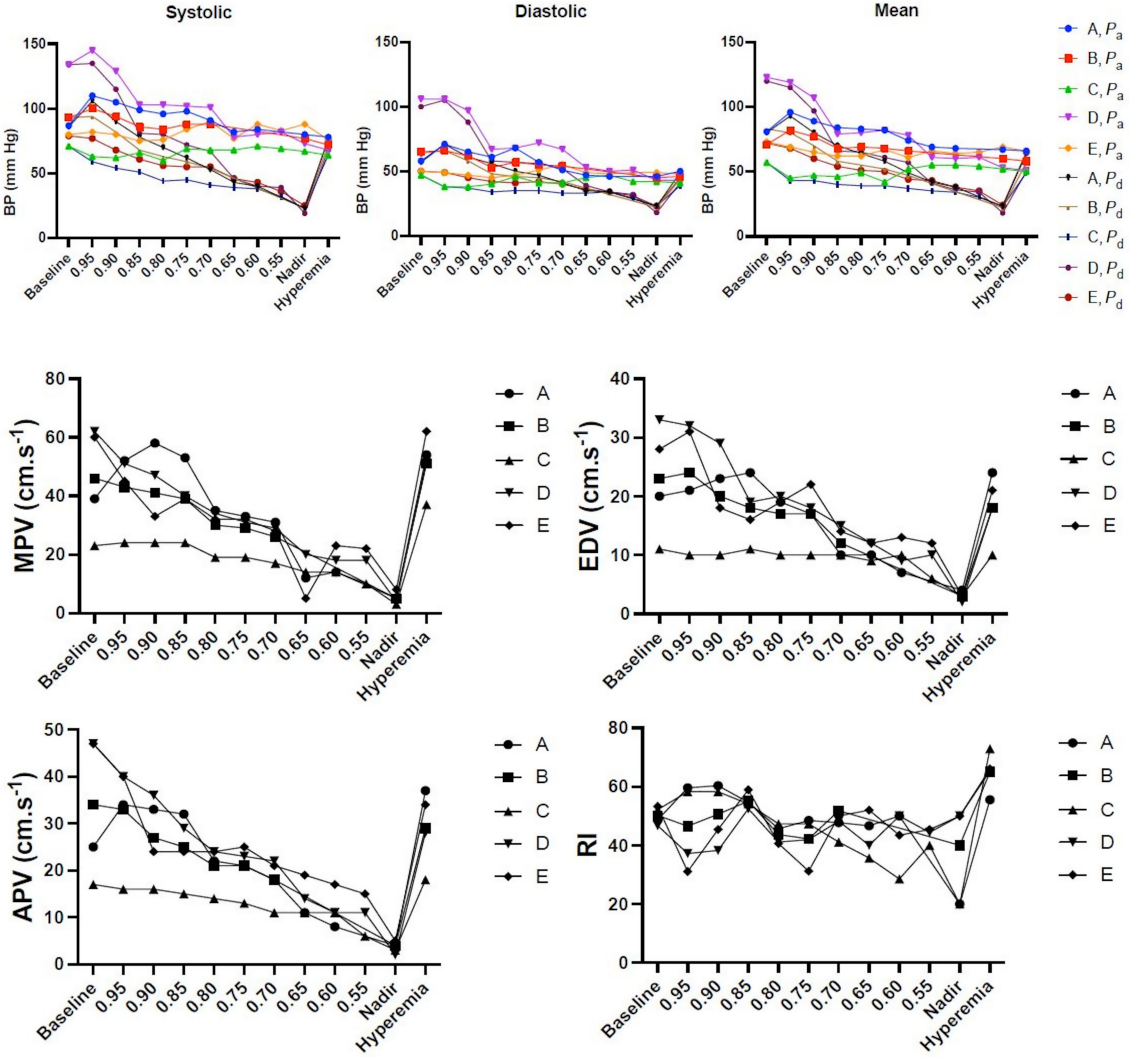
Evolution of hemodynamic parameters. Top panel depicts the evolution of systolic (left panel), diastolic (middle panel), and mean (right panel) blood pressure of the five animals which developed a significant renal artery stenosis 6 weeks after clipping. A, B, C, D and E each represent the value of an individual animal. Data are given for aortic pressure (*P*_a_) and for the pressure distal to the graded stenosis inflicted by progressive balloon inflation (*P*_d_). *x* axis depicts the different conditions during which measurements were obtained: Baseline; during progressive ballon inflation to induce a graded stenosis based on the ratio between *P*_d_ and *P*_a_ in 5% decrements; nadir represents the lowest *P*_d_/*P*_a_ ratio reached; hyperemia is after balloon deflation. *y* axis shows the blood pressure values (BP) in mm Hg. Lower panel presents the renal flow parameters for each individual animal (A–E, the letters reflect the same animal throughout the graph). Values are shown for maximum peak velocity (MPV), end‐diastolic velocity (EDV), average peak velocity (APV) in cm.s^−1^, and for resistive index (RI); these values are shown on the *y* axis. *x* axis represents the different conditions during which the measurements were obtained: Baseline; during progressive balloon inflation to induce a graded stenosis based on the ratio between *P*_d_ and *P*_a_ in 5% decrements; nadir represents the lowest *P*_d_/*P*_a_ ratio reached; hyperemia is after balloon deflation.

Average peak velocity (APV) remained relatively constant up to a *P*_d_/*P*_a_ ratio of 0.80. However, once the *P*_d_/*P*_a_ ratio became <0.80, APV decreased significantly from 34.0 ± 15.4 to 21.0 ± 4.8 cm.s^−1^ (Figure 5).

A perfusion pressure decrease ≥20% translated into a significant decrease in RI (from 50.3 ± 3.0% to 43.7 ± 3.3%; *p* = 0.05). RI reached its lowest value at the distal pressure nadir during maximal balloon inflation and normalized rapidly after complete balloon deflation (Figure 5).

### 3.7 Effect of graded RAS on neurohumoral parameters in the presence of *contralateral* significant RAS

A significant increase of renin levels was observed. Ipsilateral right venous renin augmented from 66.3 ± 24.6 to 103.1 ± 49.7 pg.mL^−1^ at a *P*_d_/*P*_a_ ratio of 0.70; *p* < 0.05.

A similar increase in arterial and in left venous renin was observed from 66.7 ± 24.5 and 66.1 ± 23.4 pg.mL^−1^ to 100.3 ± 50.4 and 101.8 ± 54.4 respectively at the 30% decline in perfusion pressure.

**FIGURE 6:**
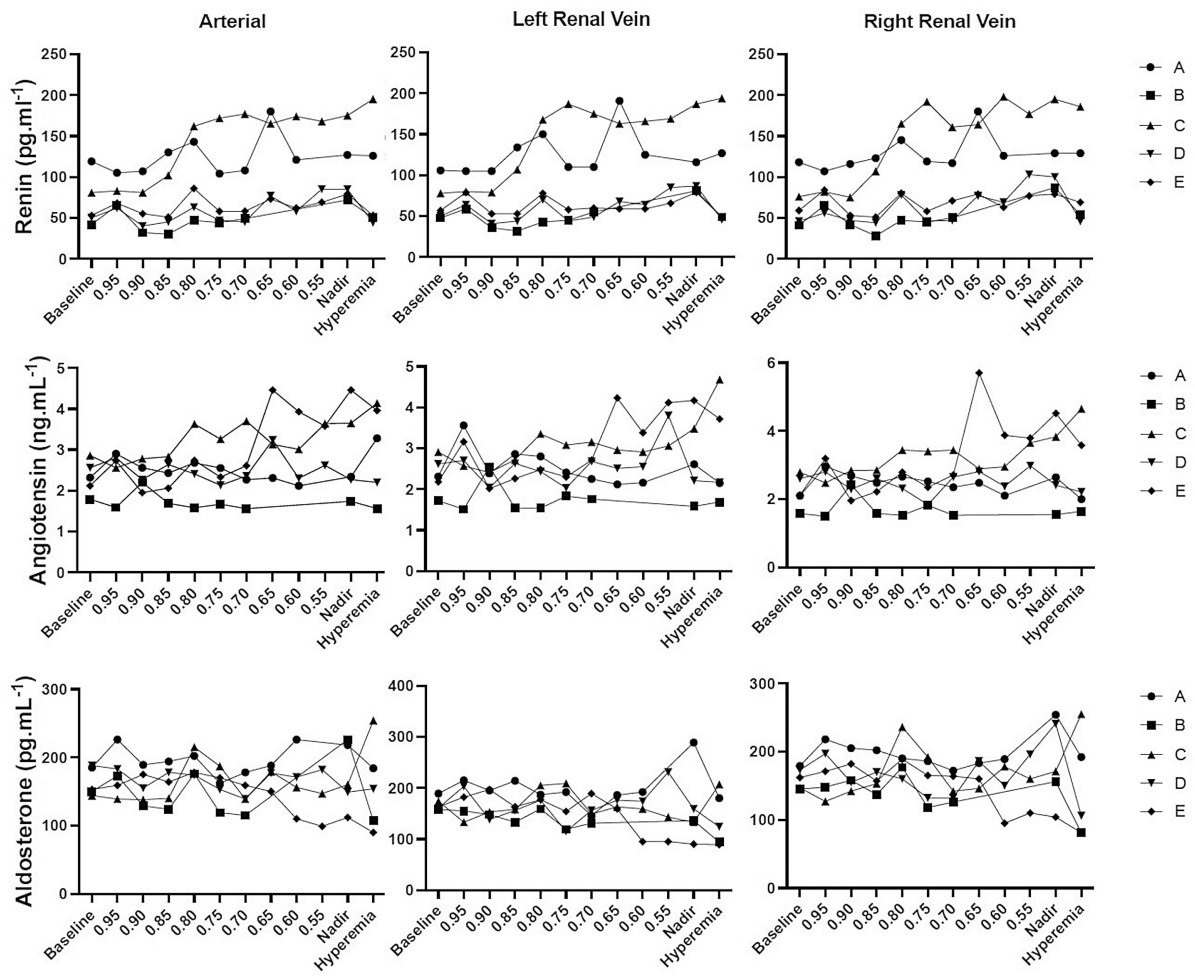
Evolution of neurohumoral parameters. Individual data are represented for each animal (A–E, each letter reflects the same animal throughout the graph) for renin (top panel), angiotensin (middle panel), and aldosterone (lower panel) in the aorta (arterial, left panel), left renal vein (middle panel), and right renal vein (right panel). Details on the methodology for renin, angiotensin, and aldosterone: See methods section. *x* axis represents the different conditions during which the measurements were obtained: Baseline; during progressive balloon inflation to induce a graded stenosis based on the ratio between *P*_d_ and *P*_a_ in 5% decrements; nadir represents the lowest *P*_d_/*P*_a_ ratio reached; hyperemia is after balloon deflation. The *y* axis denotes the values for respectively renin (top panel, in pg.mL^−1^), angiotensin (middle panel, in ng.mL^−1^) and aldosterone (lower panel, in pg.mL^−1^).

No significant changes in Angiotensin or Aldosterone levels were observed (Figure 6).

### 3.8 Comparison of renin activation during graded RAS in the presence or absence of contralateral RAS

**FIGURE 7:**
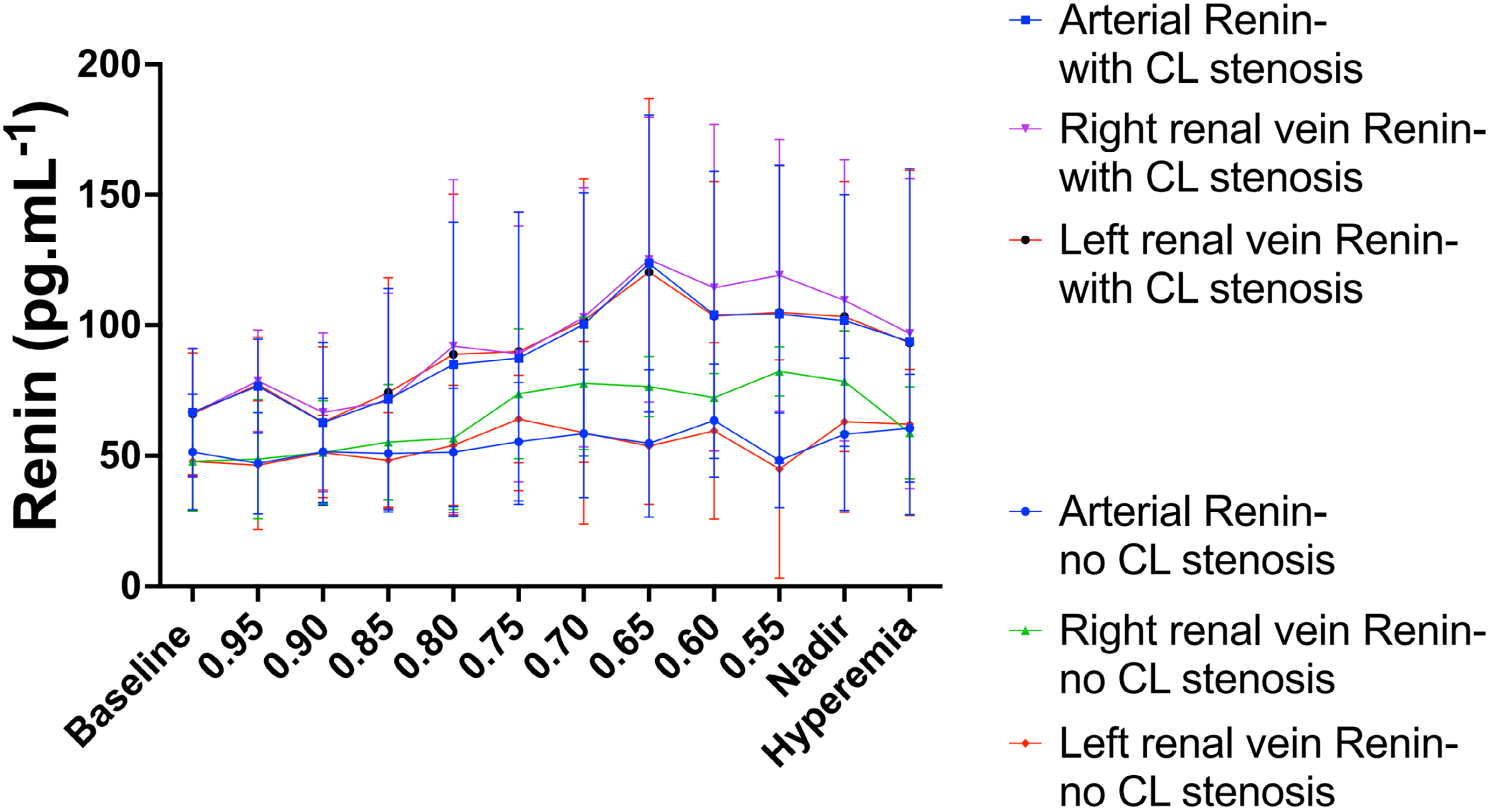
Comparison of Renin concentrations. Comparison of Renin levels 6 weeks after the index experiment of the pigs that developed a significant stenosis, defined as a ratio between distal and aortic pressure (*P*_d_/*P*_a_) ≤0.75 (“with CL stenosis”) after clipping of the *left* renal artery (*n* = 5) with those that did not develop a significant (*P*_d_/*P*_a_ ratio >0.80) stenosis after clipping (“no CL stenosis”) (*n* = 7). Values are expressed as mean ± SD. Renin samples were obtained in the aorta (“arterial”) and in both renal veins (left and right renal vein resp). Details on the methodology for renin: See methods section. *x* axis depicts the different conditions during which measurements were obtained: At baseline condition and during induced graded renal artery stenosis in the *right* renal artery by progressive balloon inflation; stenosis severity is expressed as the ratio between distal renal pressure (*P*_d_) and aortic pressure (*P*_a_). Nadir denotes the lowest *P*_d_/*P*_a_ ratio. Hyperemia is reached after balloon deflation. *y* axis represents renin values (in pg.mL^−1^).

We compared renin activation during graded RAS during the index experiment (with a normal left renal artery) with renin values during graded RAS in the presence of a significant contralateral (left) RAS. Although not statistically significant, a clear trend towards more pronounced renin activation was observed in the presence of contralateral RAS (Figure 7).

## 4 DISCUSSION

In an animal model of unilateral graded RAS in the presence of a significant contralateral stenosis, a 25% decrease in perfusion pressure results in a significant decrease in distal renal flow, causing a more pronounced upregulation of renin secretion when compared to a model of graded unilateral RAS without significant contralateral stenosis.

In literature, many experimental models are described to study renovascular hypertension. Goldblatt, seeking for an experiment to mimic the pathophysiological changes found in human essential hypertension, was the first to describe a canine experiment to induce arterial hypertension by clipping one renal artery while keeping or surgically removing the contralateral kidney (Goldblatt et al., 1934).

In the 1K1C model, RAS is created at one side and the contralateral kidney is surgically removed. An expansion of plasma volume is observed because of sodium retention (Pickering, 1989). Typically, normal plasma renin levels are measured (so called “low‐renin hypertension”) (Gross, 1971; Hall, 1991; Laragh et al., 1975).

The experimental model of 1K1C is interesting from a methodological and pathophysiological point of view but its clinical counterpart is infrequently encountered.

In the 2K1C model, which clinical counterpart is a significant unilateral RAS, a rise in renin secretion is observed from the kidney supplied by the clipped stenotic artery. The renin content of the contralateral kidney is correspondingly diminished (de Jong, 1969). A rise in peripheral venous renin is also observed, however there is a closer correlation between renal vein renin from the ischemic kidney and BP elevation than between peripheral venous renin and BP (Leenen et al., 1973). Administration of RAAS blockade results in a significant decrease in BP (Brunner et al., 1971; Gavras et al., 1973).

The most logical explanation for the difference between the 1K1C and 2K1C hypertension is that in the latter case the contralateral kidney excretes sodium normally, thereby preventing sodium retention (Laragh et al., 1975).

It is recognized that many patients with unilateral stenosis have subclinical contralateral disease, rendering the 2K1C model not fully representative for the clinical model of “unilateral” RAS. An autopsy series demonstrated that 60% of patients with a severe stenosis of one renal artery had a severe stenosis of the contralateral side. Only 10% of patients had a completely normal contralateral renal artery (Schwartz & White, 1964). Therefore, it is important that the same cut‐off values to define hemodynamic significance of a given RAS can be applied irrespective of contralateral renovascular disease.

Bilateral RAS is frequently encountered in clinical practice. In a series of 72 patients undergoing surgery for RAS, 25 patients had bilateral stenosis (Hansson et al., 1981). Its experimental counterpart would be the 2K2C model but data are surprisingly sparse. Yamasaki (Yamasaki, 1987) performed an elegant experiment in rabbits. First, one kidney was clipped resulting in a 2K1C model and after 2 weeks, the other renal artery was clipped. Both BP and plasma renin activity were increased in the 2K1C model. However, after the second clip BP further increased while plasma renin activity returned to normal. Thereafter, when one kidney was removed, there was a further elevation in BP with no further change in plasma renin activity.

Our data show that in bilateral RAS, the same cut‐off values to define hemodynamic significance of RAS can be applied as in unilateral stenosis, which is important in the clinical work‐up of bilateral RAS, which is frequently encountered in clinical practice.

We chose to define significant RAS as a function of pressure gradient as it is easily measured and as it unequivocally demonstrates that a given stenosis induces a pressure gradient. In a previous experiment we demonstrated that a pressure gradient of >25% (*P*_d_/*P*_a_ radio ≤0.75) induces a decrease in renal perfusion pressure and an increase in renin secretion (Drieghe et al., 2023). Six weeks after clipping the left renal artery, baseline flow parameters in the right renal artery were higher and there was a clear increase in BP, however not statistically significant, most probably due to the limited number of subjects.

Baseline RI was lower in the presence of a contralateral significant stenosis which might indicate a lower peripheral resistance to compensate for the impaired contralateral renal perfusion.

During induction of graded RAS in the presence of a contralateral significant RAS, a more pronounced rise in renin secretion in the ipsilateral renal vein was observed as compared to the findings in graded RAS with a healthy contralateral renal artery. Furthermore, we noted an increase in contralateral venous and arterial renin, which was not documented in the previous experiment (Drieghe et al., 2023). This might suggest that compensatory mechanisms are more addressed in the presence of a contralateral RAS.

In our model, a nonsignificant increase in BP and renin levels was observed. This might be explained by the limited number of subjects developing significant RAS after clipping. Another explanation might be that after 6 weeks, renin levels already returned to close‐to‐baseline levels. In contrast to Samani et al., 1989, we did not observe a lateralization in favor of the clipped stenotic kidney.

It is well recognized in animal models that placement of a vascular clip does not always result in sufficient and reproducible luminal narrowing. Success rates of 44–80% are described in rats and of 45% in mice (Chelko et al., 2012). Despite standardized placement of 2 adjacent titanium vascular clips, which were not dislodged after the index experiment as documented on fluoroscopy, in only five animals a hemodynamically significant stenosis was observed, defined as a translesional (“trans‐clip”) pressure gradient of ≤0.75.

### 4.1 Limitations

While gaining important insights on renal pressure‐flow relation and neurohumoral activation, we acknowledge that in this experiment in healthy animals, a kidney with normal arterial inflow is acutely forced to activate its compensatory systems. In humans, atherosclerotic RAS is a chronic vascular disease whereby a significant stenosis develops gradually over a longer time before reaching hemodynamic significance. One may assume that other mechanisms protecting the renal function are interfering with this condition (Schreiber et al., 1984; Zierler et al., 1994).

## 5 CONCLUSION

In a porcine model of unilateral graded RAS in the presence of a significant contralateral stenosis, a 25% decrease in perfusion pressure results in a significant decrease in distal renal flow, causing a more pronounced upregulation of renin secretion when compared to a model of graded unilateral RAS without significant contralateral stenosis.

These data show that in bilateral RAS, the same cut‐off values to define hemodynamic significance of RAS can be applied as in unilateral RAS, which is important in the clinical work‐up of bilateral RAS. Integration of renal hemodynamic data in the equation of management of RAS might improve patient selection for renal artery intervention, thereby avoiding unnecessary intervention and improving patient outcomes. Therefore, future trials on renal artery interventions should include transstenotic pressure gradient measurements.

## AUTHOR CONTRIBUTIONS

Conceived and designed research: BD, MDB, TDB. Performed experiments: BD, TB. Analyzed data: BD, SS, GVL. Interpreting results: BD, GVL, MDB, TB, TDB. Preparation of figures: BD, MDB. Drafting manuscript: BD, MDB, TB, TDB. Editing and revising manuscript: BD, GVL, MDB, TB, TDB. Approving final version of manuscript: BD, GVL, SS, MDB, TB, TDB.

## FUNDING INFORMATION

No funding information provided.

## CONFLICT OF INTEREST STATEMENT

None.

## ETHICS STATEMENT

The study complied with the standards of “the guide for the care and use of laboratory animals” published by the National Institute of Health (National Research Council (US) Institute for Laboratory Animal Research, 1996) and was approved by the institutional ethics committee for animal research (Ghent University Hospital, ECD 12/11).

## Data Availability

The data are available from the authors upon request.
